# Autonomous Small-Angle Scattering for Accelerated Soft Material Formulation Optimization

**DOI:** 10.1021/acs.chemmater.5c00860

**Published:** 2025-06-06

**Authors:** Tyler B. Martin, Duncan R. Sutherland, Austin McDannald, A. Gilad Kusne, Peter A. Beaucage

**Affiliations:** † Materials Science & Engineering Division, 10833 National Institute of Standards and Technology, Gaithersburg, Maryland 20899, United States; ‡ NIST Center for Neutron Research, National Institute of Standards and Technology, Gaithersburg, Maryland 20899, United States; § Materials Measurement Science Division, National Institute of Standards and Technology, Gaithersburg, Maryland 20899, United States

## Abstract

The pace of soft material formulation (re)­development and design is rapidly increasing as both consumers and new legislation demand products that do less harm to the environment while maintaining high standards of performance. To meet this need, we have developed the Autonomous Formulation Lab (AFL), a platform that can automatically prepare and measure the microstructure of liquid formulations using small-angle neutron and X-ray scattering and, soon, a variety of other techniques. Here, we describe the design, philosophy, tuning, and validation of our active learning agent that guides the course of AFL experiments. We show how our extensive *in silico* tuning results in an efficient agent that is robust to both the number of measurements and signal-to-noise variation. Finally, we experimentally validate our virtually tuned agent by addressing a model formulation problem: replacing a petroleum-derived component with a natural analog. We show that the agent efficiently maps both formulations and how post hoc analysis of the measured data reveals the opportunity for further specialization of the agent. With the tuned and proven active learning agent, our autonomously guided AFL platform will accelerate the pace of discovery of liquid formulations and help speed us toward a greener future.

## Graphical abstract

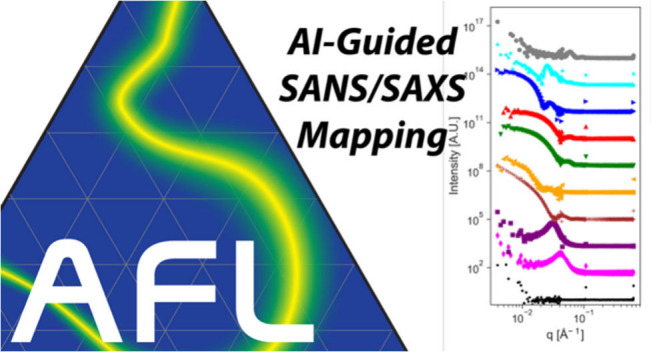

## 1 Introduction

The time scale of innovation driven by fundamental understanding in materials development is often on the order of years. For example, the key technologies for carbon-fiber reinforced polymers (CRFP) were developed in the 1950s and 1960s, yet the first passenger airplanes with significant use of CFRP only found commercial application in the 2010s – a 50-year lag. Accelerating the materials innovation lifecycle from fundamental understanding to product-on-market is crucial to meeting the challenges of energy, water, health, and environment required by the growing global population. Efforts to accelerate this lifecycle, such as the Materials Genome Initiative,^1^ have largely focused on harnessing advanced modeling and modern informatics to supplement human expertise in deciding which experiments to perform to produce the maximum measurement value from the minimum number of experiments. Such workflows and related *in silico* informatics approaches have been transformative in exploring the equilibrium phase diagrams of hard materials, such as metals and ceramics, and some nonequilibrium materials processing.

In contrast to metals and ceramics, where compositions with five elemental components are considered highly complex,^2,3^ liquid formulations regularly consist of tens to hundreds of components with the physics of structure formation driven by each component. Formulated products span the breadth of modern society, from pharmaceuticals, personal care products, and food and beverage to drilling fluids and industrial materials. Despite the success of advanced modeling and closed-loop studies in other areas, most formulations are developed using human expert intuition or, at best, design-of-experiments (DOE) strategies focused on phenomenological or structural properties. This gap originates in several fundamental features of formulations: they are nearly always highly multicomponent, their structures are often far-from-equilibrium with significant processing pathway dependence, and their structure–property relationships are frequently highly complex and poorly theoretically understood. Take, as an example, a typical hair shampoo. At its core, the product is a simple ternary mixture of surfactant, oil (such as conditioners, fragrances, etc.), and water. However, the real formulation almost always includes a blend of several ionic and nonionic surfactants (e.g., multiple sodium laureth sulfates (SLES) with different ethylene oxide unit lengths or cocamidopropyl betaine (CAPB)), the oil component includes tens to hundreds of different species with diverse chemical functionality and physics, and the water component includes salts and pH adjusters. These components can interact to produce a variety of different microstructures from spherical and wormlike micelles to vesicles, which in turn produce a given final property, such as viscosity based on micelle entanglement or other macromolecular factors. In such a complex landscape, small changes in single components (for example, changing which fragrance blend is used or the overall fragrance loading) can result in unexpected changes in final properties. It is extraordinarily difficult to know in advance how far a given formulation is from a performance boundary and the potential impact from a minor variation in a manufacturing parameter. Fundamental structural understanding can be transformative to this process, but we generally lack physical theories capable of handling these complex formulation systems with sufficient accuracy.

To address this complexity, we have developed a flexible and open automation platform, the Autonomous Formulation Laboratory (AFL), which is capable of automated preparation and measurement of liquid formulations via pipetting. It can be coupled to small-angle X-ray or neutron scattering instruments for structural ‘ground truth’ measurements together with benchtop performance data such as UV–vis, turbidity, and capillary viscometry.^4^ While this platform provides enhanced reproducibility and throughput for measurements, the realization of its full potential for accelerated materials discovery requires data approaches that can, with minimal human input or training data, accurately ***interpret/label*** measurements, ***extrapolate*** those measurements into a statistically derived phase diagram, and ***choose*** which next measurement to perform toward a specific scientific objective, such as determination of the desired phase boundaries, the discovery of the overall phase behavior and boundaries of an unknown system, or the optimization of a property of interest. The difference between an automated platform and an autonomous one is the decision-making *agent* that optimally guides the course of an experiment.

For hard materials science and small molecule chemistry, agent-guided closed-loop autonomous experiments have shown great value in discovering new materials, optimizing material properties, and mapping phase boundaries.^5–12^ Many of these studies have moved past the simple application of black box machine learning techniques and have attempted to incorporate material or measurement physics into their agents. This can include incorporating thermodynamic constraints (e.g., Gibbs phase rule), knowledge of crystallographic concepts like peak shifting, or knowledge about the potential mathematical descriptions of physical phenomena.^5,8,12^ Others have sought to add explainability to their agents, such as through interpretable constraints applied to latent spaces of known variables.^13^ Frameworks have been developed for allowing multiple autonomous agents to interactively collaborate.^14^ Additionally, there is a growing conversation in the autonomous experimentation community on how to implement “human-machine teaming” concepts where autonomous agents collaborate with humans to combine the speed of autonomous agent decision making with human knowledge and intuition.^15–18^

Comparatively, the application of active learning and autonomous techniques to polymer and soft material systems is less developed. Several groups have identified reversible addition–fragmentation chain transfer (RAFT) polymer synthesis as being highly amenable to flow geometries and therefore automation.^19–22^ These studies sought to optimize material properties by tuning the polymer synthesis to control the polymer sequence or molecular weight distributions of their polymers. There have been a smaller number of studies on polymer property optimization of polymer formulations.^23–26^ These studies optimize material properties by tuning composition rather than synthesis. Of particular interest are studies that take industrial interests into account, such as the cost of the formulation chemistry.^26^

Here we report the development of a modular active learning agent that leverages small-angle scattering (SAS) measurements for phase discovery. Our agent is designed to be general-purpose such that it can be applied to scattering (and nonscattering) instruments and a range of material systems without prior knowledge of a system’s phase behavior. In the following sections we discuss the design of the agent, our *in silico* tuning approach, and finally closed-loop experimental validation delivering a performance increase of as much as 25x compared to naïve grid searches.

## 2 Agent Architecture

### 2.1 Label Data

Figure 1 is a schematic depiction of our agent’s active learning process with the full details found in Section S1 of the Supporting Information. Here we provide an overview of the agent’s design.

**Figure 1:**
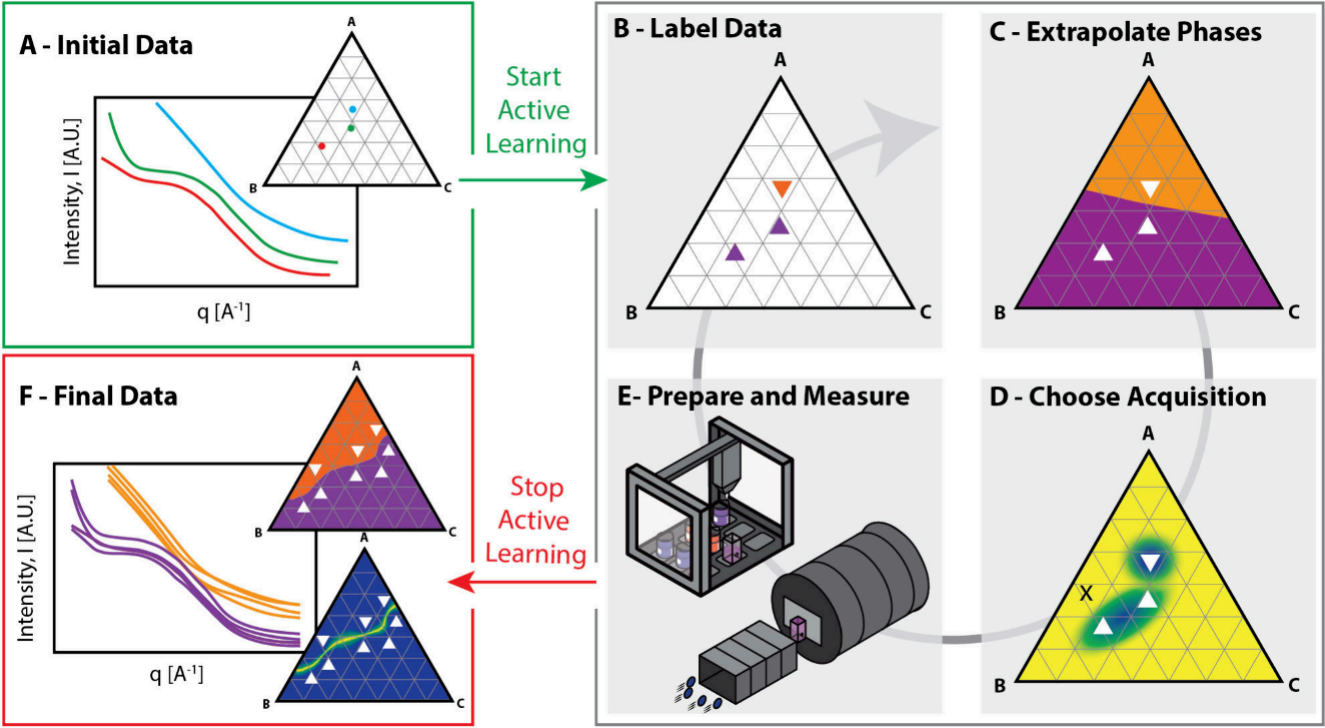
Schematic overview of the active learning agent. (A) A phase mapping problem is posed to the agent in the form of a set of least two small-angle scattering data labeled with the composition of the sample. These data can originate from expertly chosen, literature gathered, or randomly measured data. (B) These points are labeled and grouped using similarity and clustering analyses. (C) The labeled data is then extrapolated over the entire composition space using a Gaussian process classifier. (D) The next sample that best achieves the measurement goal is chosen using an acquisition function. (E) The chosen sample composition is robotically prepared and measured, after which the loop can either continue or be broken. (F) The loop is broken when the measurement goal is achieved with sufficient confidence from the AFL operator.

The agent begins each iteration of the active learning loop by analyzing the current scattering data and assigning each pattern to a discrete phase group. Even for highly trained *human* practitioners, interpreting SAS data is a highly nontrivial task. Due to the inherent heterogeneity of soft materials and the nature of SAS measurements, many microstructures can produce the same or similar scattering patterns. Choosing from the plethora of available geometric, thermodynamic, and empirical models requires specialized knowledge of the system under study, and often the results of other non-SAS measurements. Even with an appropriately chosen model, fitting SAS models can be challenging and perfect fits to experimental data are rare due to nonidealities in the instrument and sample.

The most direct approach to automating SAS data analysis would be to develop a classifier that identifies one or more appropriate analytical models, fits those models, and then uses the fit parameters to identify phases. While high-performing ML classification models for SAS exist in the literature, they have largely been applied to theoretical data and, in many cases, are limited to predicting specific sets of models which might not cover all use cases.^27–31^ To overcome this challenge, we leverage a SAS-model-free approach that combines similarity analysis with a clustering routine that gathers the data into groups of similar scattering. This group identity of each measurement acts as the phase label for that composition, i.e. all data in cluster 1 belong to “phase 1”. While this similarity approach to phase identification does not give the true structural label of the phase (e.g., spherical micelles or lamellae) it does not require *a priori* knowledge of the phase behavior of a system before beginning a campaign.

The core of this approach lies in a mathematical similarity kernel. Tuning both the form and parameters of this kernel is necessary to tailor the autonomous agent to SAS measurements. Identifying a common similarity kernel across material systems is analogous to coming up with a generalized taxonomy for SAS data: in some contexts, scattering data should be sorted into “crystalline” and “amorphous” while other data sets might demand differentiation between amorphous states (cylindrical particles vs spherical, etc.). To identify candidates for generally applicable similarity kernels, we developed a virtual instrument platform that allowed us to screen >250 000 labeling pipelines and identify the best candidates for application. The code used to generate the database of labeling results can be found in Reference 32. As described in Section 3, we find several pipelines that provide accurate labeling but are also robust to various measurement details (e.g., number of data points, measurement noise).

This similarity approach represents a highly general and adaptable method for analyzing and labeling SAS data. While we have focused on SAS, nothing in the above method is strongly specialized for this class of measurement, and the similarity approach is likely generalizable to almost any form of single-dimensional data. In multidimensional data, such as grazing-incidence wide-angle X-ray scattering (GIWAXS) data, it is likely that the similarity approach may struggle to distinguish physically relevant changes from other changes, and input preprocessing might be necessary to e.g. standardize or infill pixel gaps.^33^ On the other hand, physically informed slicing of such data, e.g. the Yoneda band or radial sectors, would likely enable drop-in use with the present pipeline. It is straightforward to test similarity metrics and clustering accuracy on premeasured data, so adapting the featurization/labeling steps to a new measurement (e.g., spectroscopy or rheology) is not challenging. In addition, the similarity approach leaves an easy route toward incorporating multiple measurement techniques by combining similarities from multiple sources.

### 2.2 Extrapolate Phases

Once the data set is labeled, the next step is to extrapolate the labels from the specific compositions at which they were measured as shown in Figure 1C. To accomplish this, we fit a variational Gaussian process (GP) classifier to the labeled data. The details of our GP implementation and optimization process can be found in Section S1.2 of the Supporting Information. From the optimized GP, we evaluate 2 functionals for each of the *N* phases identified in the previous step: the mean, *μ*_*i*_ (x*), which represents the probability of phase *i* existing at any (measured or unmeasured) composition x* and the posterior uncertainty, *σ*_*i*_ (x*), which is the variance in *μ*_*i*_ at x*. Using these functions, we can produce composition maps that, at every composition, identify the most likely phases and our overall confidence in that prediction.

### 2.3 Choose Acquisition

The final analysis step of the agent is to choose the next sample composition for measurement that best accomplishes the campaign’s goals. Examples of campaign goals include mapping all phase boundaries of a system, mapping those of a specific phase, or optimizing a physical property calculated from the SAS data or other measurement. In the active learning community, acquisition functions are typically described as combining the characters of exploitation (trusting and leveraging the current surrogate model) and exploration (searching outside of already sampled regions). For a typical task of identifying all phase boundaries, we choose to measure at the points of highest variance which is typically characterized as “pure exploration”. The approach is grounded in the fact that the GP’s uncertainty is maximized when the probability of multiple phases existing at a point is equal i.e. a phase boundary. We make three changes to the classic pure exploration approach: (1) We regularly measure poorly sampled regions in composition space, (2) we ensure that no measurements are chosen too close to one another, and (3) we randomly sample from the top 5% to 10% of variance values rather than choosing the singular true maximum value.

We choose to employ this “super exploration” since, in both *in silico and* experimental studies (*vide infra*), we found a series of edge conditions where the combination of the experimental data, classification parameters, and GP kernel tuning would produce a very stable GP solution with maximum uncertainty at a single point, put more simply an insatiable drive to remeasure the same region of phase space. Such points often seem to arise from either unhandled experimental error states (e.g., a sample which does not reach the measurement cell despite safeguards) or from exploring regions of the phase diagram with large changes in scattering that are not of relevance to the problem being studied (e.g., measuring surfactant-free samples in a surfactant phase diagram, which will have almost no scattering). To mitigate this in the context of extended runs without human guidance, we incorporated a “periodic random step”; every *n* iterations of the active learning loop (where *n* is between 3 and 10), the input uncertainty function is replaced with a random field. As a result, due to the restriction on the closeness of measurements referenced above, the system samples a random region of phase space with low measurement density. We find this approach highly effective in avoiding an extended focus on fixed local maxima of uncertainty and providing more reliable unattended sampling, especially in the context of the early stages of a measurement campaign where the agent might otherwise engage in a “depth-first” search, fully mapping the bounds of known phases before discovering others.

To further stabilize the system against such problems, we select our next measurement from the maximum uncertainty in a slightly unconventional way: we select all points from our fixed calculation grid with uncertainty within a certain percentage of the maximum, typically the top 5% to 10% of uncertainty, and then uniformly sample from this set. In doing this, we effectively buffer the measurement engine from numerical oddities of the GP solve, while still making quantitative use of the uncertainties generated. An alternative to this fixed cutoff would be to use an optimization method (e.g., a deflationary method) to identify multiple maxima in the acquisition function and choose among those maxima rather than from all points above a cutoff; this approach could produce better distributed sampling at the cost of complexity, but may be of limited use in the context of a fixed sampling grid.

This acquisition function approach has proven to be both stable and produce trusted results. Furthermore, it is highly adaptable and can be tuned for specific tasks (e.g., boundary identification of a specific phase or cost optimization) or to incorporate material nonidealities or instrumental effects (e.g., hysteresis or motor movement time). For the full details of our implementation, see Section S1.3 of the Supporting Information.

## 3 *In Silico* Agent Tuning

A central challenge in the application of autonomous approaches to experimental data is the availability of high-quality reference data with realistic artifacts, noise, and other features for training and benchmarking of classifiers, tuning of GP kernel parameters, and acquisition function selection. Before deploying the agent on the AFL platform, we tested, tuned, and benchmarked its performance against *in silico* experiments.

For these experiments, we sought to design a set of tests that would provide a significant challenge to the agent and allow us to benchmark it in a challenging scenario. We identified the phase diagram in Reference 34 as having a large number of phases with complex boundaries, while also being of a material class directly relevant to the AFL program and our stakeholders. We chose analogous SAS models (Figure 2A) for each of the phases (Figure 2B) in Reference 30 and, to ensure that the tuned agent would be performant on real data, we introduced experimental artifacts into the synthetic data using real measurements as templates. This includes resolution smearing, statistical noise, and artifacts from stitching data from multiple instrument configurations together consistent with data from the 10 m SANS instrument at the NIST Center for Neutron Research. See Section S2 of the Supporting Information for the full details on this process. Furthermore, we introduce *q*-dependent counting noise into the data (Figure 2C) that is based on the reference measurements but can be amplified or diminished by a noise level scale factor, η. Given the phase boundaries and reference measurements, this virtual instrument will identify the ground truth phase based on requested composition and produce a scattering pattern that can be analyzed by the agent. In this way, our agent can query the virtual instrument, add the resulting scattering pattern to its measurement corpus, and then complete a virtual closed loop by predicting and requesting a measurement at the optimal next composition.

**Figure 2:**
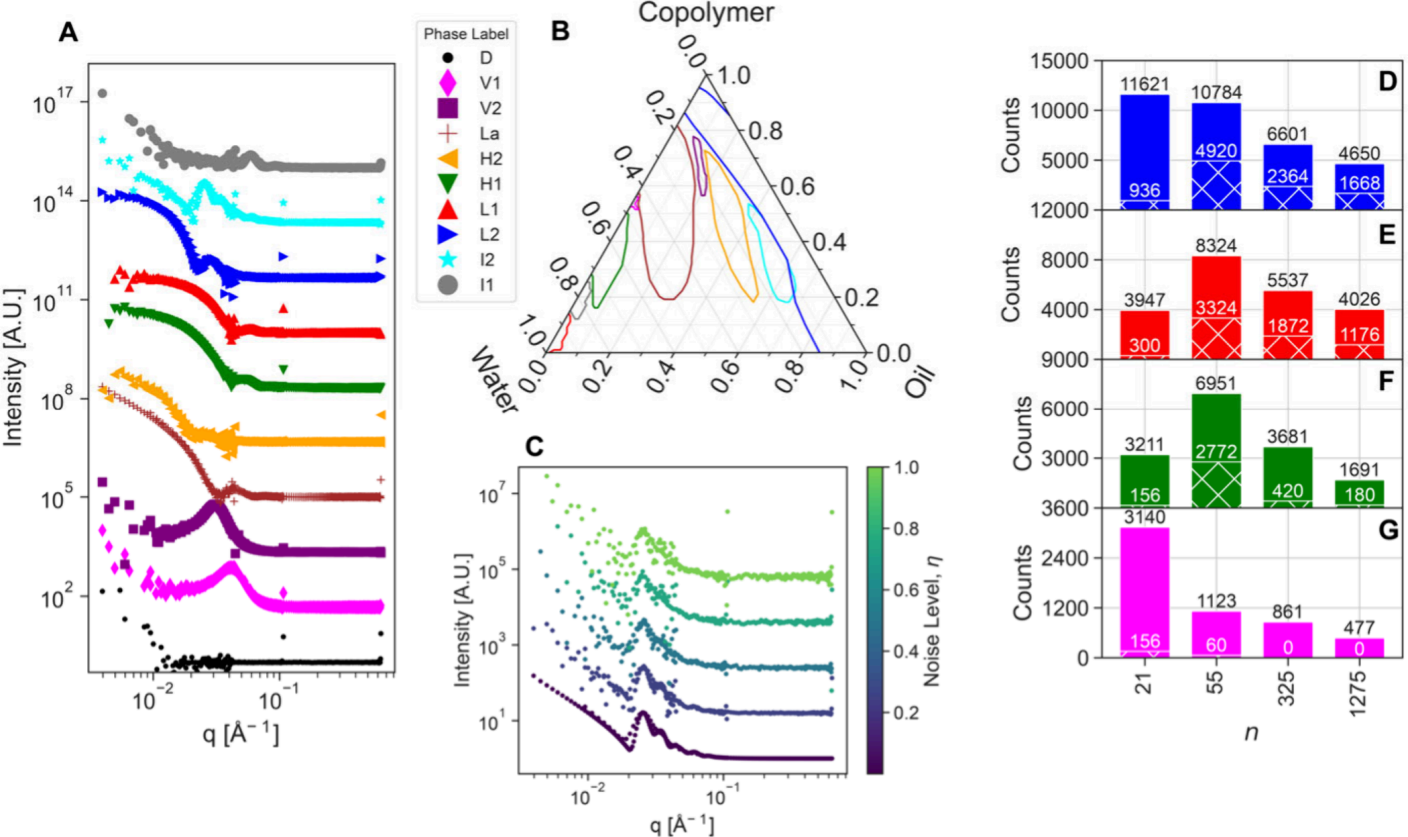
Summary of *in silico* tuning results. (A) SAS data for each phase shown in (B) are generated using a scheme that incorporates instrumental smearing and tunable counting noise (C). (D–G) The number of labeling pipelines, out of >250,000 considered, which produce FMS *≥* 0.85 (D), 0.9 (E), 0.95 (F), and 1.0 (G) for varying numbers of measurements, *n*. The ground truth phase diagrams for each *n* is shown in Figure S3. The hatched bars represent the number of pipelines that meet that subplot’s FMS criteria at all noise levels rather than being considered individually.

We break the agent tuning into two parts. Given the significant challenges associated with the labeling step of the agent, as discussed in Section II.A, we first focus on identifying a robust labeling pipeline. Our goal is to ensure that the labeling pipeline is robust at both small and large numbers of measurements, so we tested against symmetric grids with varying number of measurements, *n*, as shown in Figure S3. We then constructed over 250,000 labeling pipelines by varying both the clustering method and the form and coefficients of the similarity matrix calculation (See Section S1.1.2 of the Supporting Information). We quantify the performance of each pipeline using the Fowlkes-Mallows score defined as

1 $$FMS=\frac{TP}{\sqrt{\left(TP+FP\right)\left(TP+FN\right)}}$$

where TP refers to a true positive labeling, FP is false positive, and FN is false negative. FMS varies between 0 and 1 with 1 being a perfect match to the ground truth and 0 being a perfectly incorrect labeling. Figure 2D-G shows the number of labeling pipelines that scored FMS ≥ 0.85 (D), 0.9 (E), 0.95 (F), and 1.0 (G) as a function of *n*. As expected, as we increasingly constrain the performance of the agent going from D-F, we see the number of pipelines that satisfy the constraint decreases. This is particularly true when we consider the agents performance across different noise levels η (hatched bars) rather than individually (solid bars). For the η constrained case, we see that no agents have a perfect score, FMS = 1.0, at all *n* and all η. We find that the pipeline that has the highest FMS performance across all *n* and η has a minimum FMS of ≈ 0.946 and consists of a Gaussian mixture model and sigmoid similarity. While this pipeline has the most robust performance, one might want to choose an agent that has a higher FMS for a given noise level and number of measurements. In Table S1 we show a list of high performing pipelines and their parameters.

With the labeling step tuned for SANS measurements, we are ready to run full active learning campaign simulations. In Figure 3, we show the *in silico* performance of agents with different acquisition functions. Along with random-sampling and exploration (variance) based search, we show three versions of “super exploration” which use random instead of variance sampling for 5%, 10%, or 20% of iterations as labeled. We also show two versions of multiresolution grid sampling designed to mimic an experimentalist naïvely stepping through a preprepared set of samples. For grid sampling v1, we measure from 0 to 100% water at 0% copolymer, increase by a copolymer content by 5%, and then repeat until we reach 100% copolymer content. For grid sampling v2, we still measure from 0 to 100% water at constant copolymer concentration, but, instead of a constant step size in copolymer concentration, we repeatedly bisect the copolymer composition space. More details of these agents can be found in Section S3.5 of the Supporting Information. We quantify the performance of our agents both in terms of the “boundary score” (Figure 3A, 3B) and the number of phases identified by the agent (Figure 3C). The boundary score is the average distance of an agent identified boundary from its ground truth boundaries and is described in detail in Section S3.4 of the Supporting Information. We use the boundary score rather than calculating the FMS at each point in a composition grid, as the latter approach would bias the performance evaluation toward the largest phases by area. Furthermore, our boundary score metric is better aligned with our agent goal which is to accurately identify the location of phase *boundaries.* Figure 3A and 3B display the boundary score as a function of iteration (the number of measurements) for two representative phases within the challenge problem. Note that all phases are mapped simultaneously despite each plot showing the performance of mapping a single phase. The boundary score plots for all phases are shown in Figure S6. Figure 3D shows, for each agent, the average boundary score calculated over all iterations and just the final 25 iterations. Respectively, these derived values characterize the speed at which each agent correctly finds the boundary and the final accuracy of the agent identified boundaries. The mean represents a combination of the speed and accuracy of a given agent; both metrics are important to real phase mapping challenges. For example, an agent that requires several hundred steps before providing high accuracy is not necessarily better than one which improves more quickly but to a lower ultimate accuracy.

**Figure 3:**
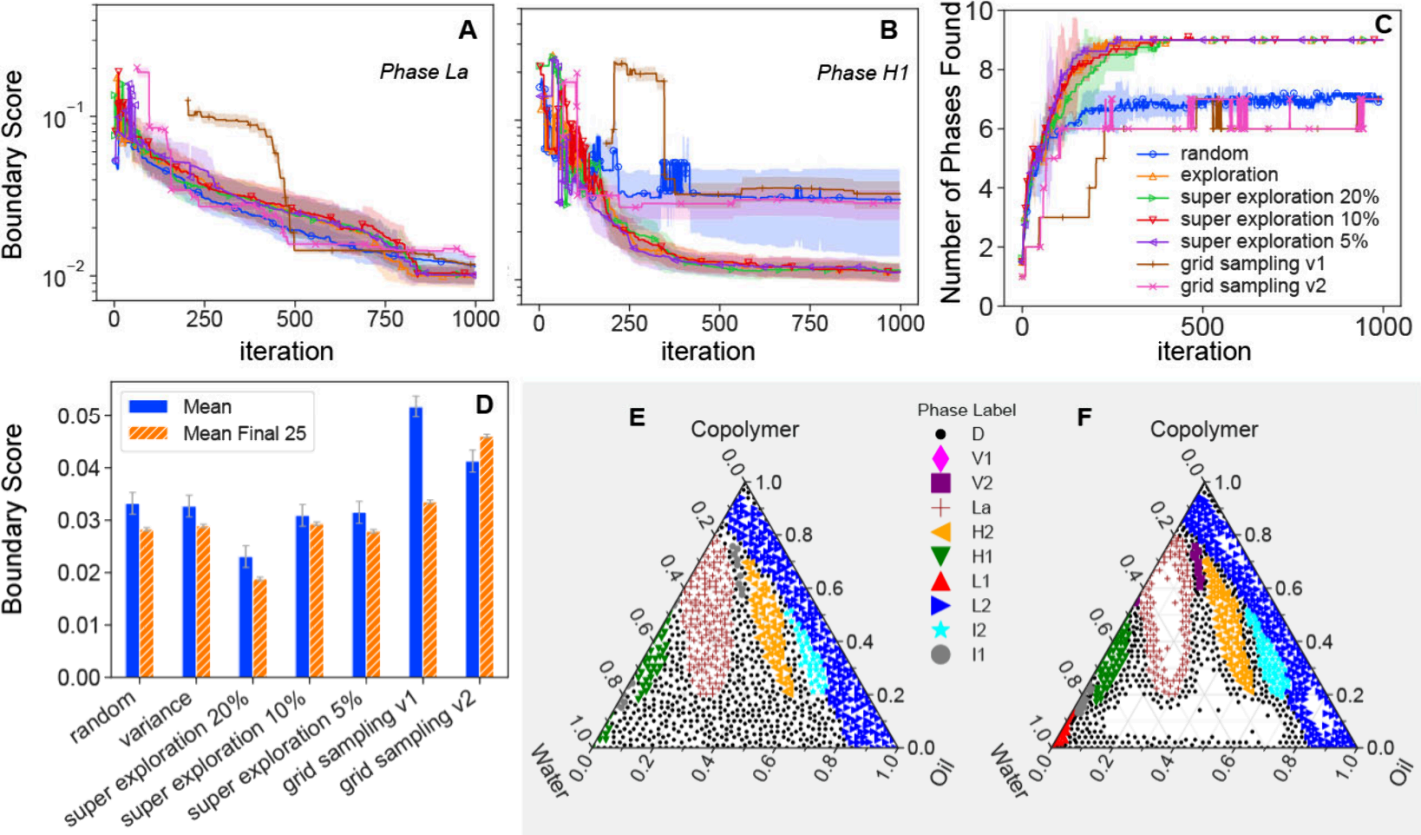
Performance of the AFL agent with full *in silico* active learning runs. (A, B) Boundary score (a metric for phase mapping performance with a lower score representing a more accurate phase map, see Section S3.4 of the Supporting Information) as a function of iteration (number of measurements) for the (A) La and (B) H1 phases with different acquisition functions as shown in the legend in (C). (C) Number of correctly identified phases (out of 10) as a function of iteration number for each algorithm. (D) Mean boundary score for each agent across either the entire 1000 iteration campaign (blue) or only the final 25 iterations (orange). The blue bar is indicative of the speed of the agent taking early iterations into account, while the orange indicates final mapping performance. For A–C, the lines and shaded regions represent the mean and standard deviation of seven independent active learning runs from different initial conditions. The error bars in Part D are the standard deviation across all trials, phases, and steps. (E, F) The final measured phase map for random (E) and pure exploration (F) acquisition functions.

In all cases, the exploration-based agents show superior performance to the random sampling or grid searches. Figure 3C shows that these agents locate more phases on average and, Figure 3D shows that the super exploration agent with 20% random sampling is superior to all other agents evaluated in this study in both speed and accuracy. While random sampling initially reduces the boundary score more quickly than the exploration-based agents for the largest phases (Figure 3A), the exploration-based agents show the greatest improvement at the end of the campaign and significantly better performance for the smaller phases. For the random and grid-based searches, the lack of data clustered around small phases leads to a breakdown in the labeling performance, leading to incorrect or nonidentification of phases. Finally, it is clear that the exploration-based agents spend more time measuring near phase boundaries than the random search from the ternary diagrams in Figure 3E and 3F respectively.

These *in silico* tests and tuning runs are crucial toward making effective use of limited neutron or X-ray beamtime at a user facility. In addition, they also validate the agent’s design and show that it reduces the number of measurements needed to map a phase space. A key feature of this sampling approach is that we identify the location of phase boundaries with far greater accuracy and greatly reduce the number of points needed to map a phase space when compared to a naïve grid search.

## 4 *In Operando* Demonstration

After demonstrating the AFL agent *in silico*, the next step is to validate the same agent in a live SAS experiment. This validation will not only verify the efficacy of the agent in mapping phase boundaries, but also the *in silico* approach used to tune the design and parameters of the agent. The latter is particularly important given the limited availability of neutron and synchrotron X-ray scattering beamtime. While we expect that the parameters we identified to be general, we also recognize that some experiments may require specific tuning of the agent in order to achieve optimal performance. Based on this reasoning, the following results are not intended to be a measure of top performance of the agent but rather a validation of using *in silico* tuning to achieve a reasonably performant agent with minimal excess beamtime used for tuning.

The agent is implemented in our open-source software package, AFL-agent.^35^ The agent is deployed to the AFL platform^4^ as a microservice (HTTP service local to the instrument) using the AFL-Automation APIServer architecture. This “*AgentServer*” interfaces with a custom-designed sample server (*SampleServer*) to facilitate closed-loop experimental measurements. The *AgentServer* handles the labeling, extrapolation, and acquisition function steps while the *SampleServer* is responsible for orchestrating the preparation, measurement, and cleanup of a sample. Separating the guidance agent from the *SampleServer* allows rapid debugging and tuning of agent hyperparameters such as similarity functions, GP kernel parameters, and acquisition functions during a run without interrupting measurement, which can be particularly important for long running measurements in, e.g., neutron scattering.

As a starting point, we use X-ray scattering to map the phase behavior of a nonionic copolymer surfactant formulation analogous to the one studied *in silico*: Pluronic F127, salt (NaCl), hexanes, and water. In this experimental campaign, we varied the mass fractions of F127, NaCl, and hexanes and fixed the total volume of each sample, thereby constraining the water component of the mixture. The results are shown in the ternary diagram in Figure 4A and the projection of the ternary into a binary concentration space appear in Figure 4B. The uncertainty colormap in these figures serves as a proxy for the location of the phase boundaries, with the brighter colors that indicate higher uncertainty in phase label also indicating the likely location of the structural phase transition. The agent identifies three clusters in the SAS data and we’ve labeled the identifying structure factor peaks on representative data from each of these clusters in Figure 4C. At low salt concentrations there are two populations of micelles of different radius (Figure 4D) which, as the salt concentration is increased, transition to what is consistent with hexagonal ordering (Figure 4E) and then lamellar ordering (Figure 4F). While the dependence of salt on the phase behavior of nonionic surfactants has been reported previously,^36^ our agent has rediscovered this trend independently, without prior or programmed knowledge of this phenomena.

**Figure 4:**
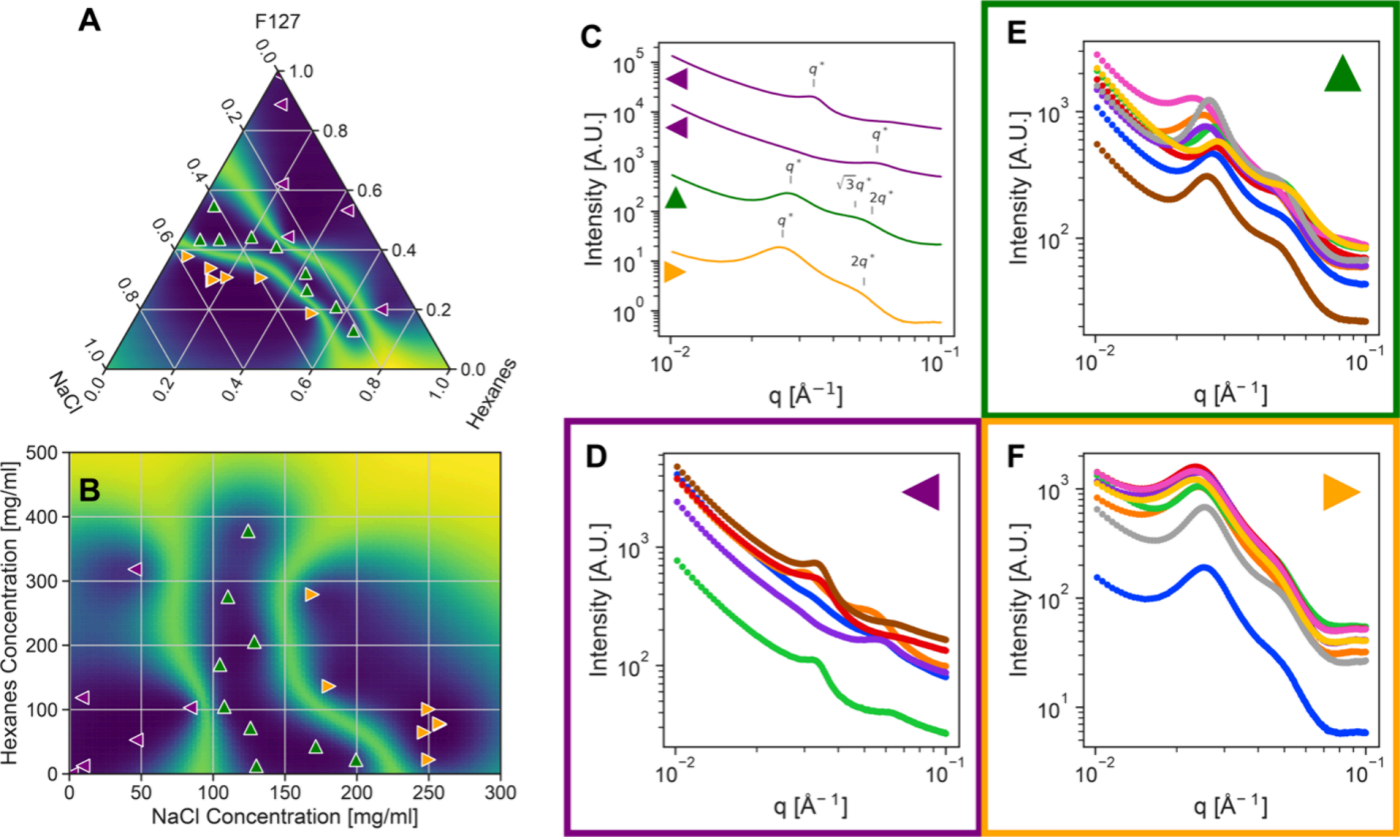
Experimental active learning results on mixtures of Pluronic F127, NaCl, hexanes, and water. (A) The final ternary phase diagram measured by the agent and (B) the binary projection of this phase diagram with the symbols denoting the phase identified by the agent and the background colormap, the uncertainty in this phase assignment. Parts D–F show the SAXS data associated with each agent identified phase. Part C shows four representative data sets with the characteristic diffraction peaks labeled indicating, from top to bottom, spherical micelles, spherical micelles, hexagonally packed cylinders, and lamellar structures.

It is important to highlight that the AFL agent performed well in this experiment without ever having been exposed to scattering data of this form. Despite our extensive attempts to benchmark the agent with “realistic” virtual instrument data, it is clear that none of the scattering in Figure 4 look quite like the data in Figure 2A. The experimental data has a strong background signal and displays Bragg scattering consistent with crystalline materials rather than the primarily “form factor” scattering that was used for the *in silico* testing. Regardless, our similarity approach to identifying data clusters allowed the agent to find regions of similar scattering within the explored composition space. While a human practitioner might have separated the two micelle structures into different phases because their sizes are different, it is entirely reasonably to construct a taxonomy that gathers the “single particle” scattering into a single grouping.

As an example of a product reformulation challenge, we took the same formulation discussed above and replaced the petroleum derived hexanes with limonene, a naturally derived analogous oil. The promise of autonomous exploration is that such challenges can be accelerated and the differences in phase behavior can be rapidly identified. We performed the same campaign with the same agent, simply replacing the component. The results are shown in Figure 5. In contrast to the hexanes system, the agent separates the micelle scattering into two groupings in the limonene system. This difference in classification likely originates from the increased number of data points we were able to gather for the limonene case. The analogous phase to the hexagonal phase observed in the hexanes case begins at a similar salt concentration but is a stronger function of the oil component concentration, with salt and limonene having a nearly equal role in driving crystalline structure formation. Similar to the hexanes system, the hexagonal phase appears at ∼ 100 g/mL NaCl and transitions to lamellar ordering at higher salt concentrations. The width of the identified phase boundaries and their multidimensional shape are critical variables for designing liquid formulations with the desired stability or engineered transitions.

**Figure 5:**
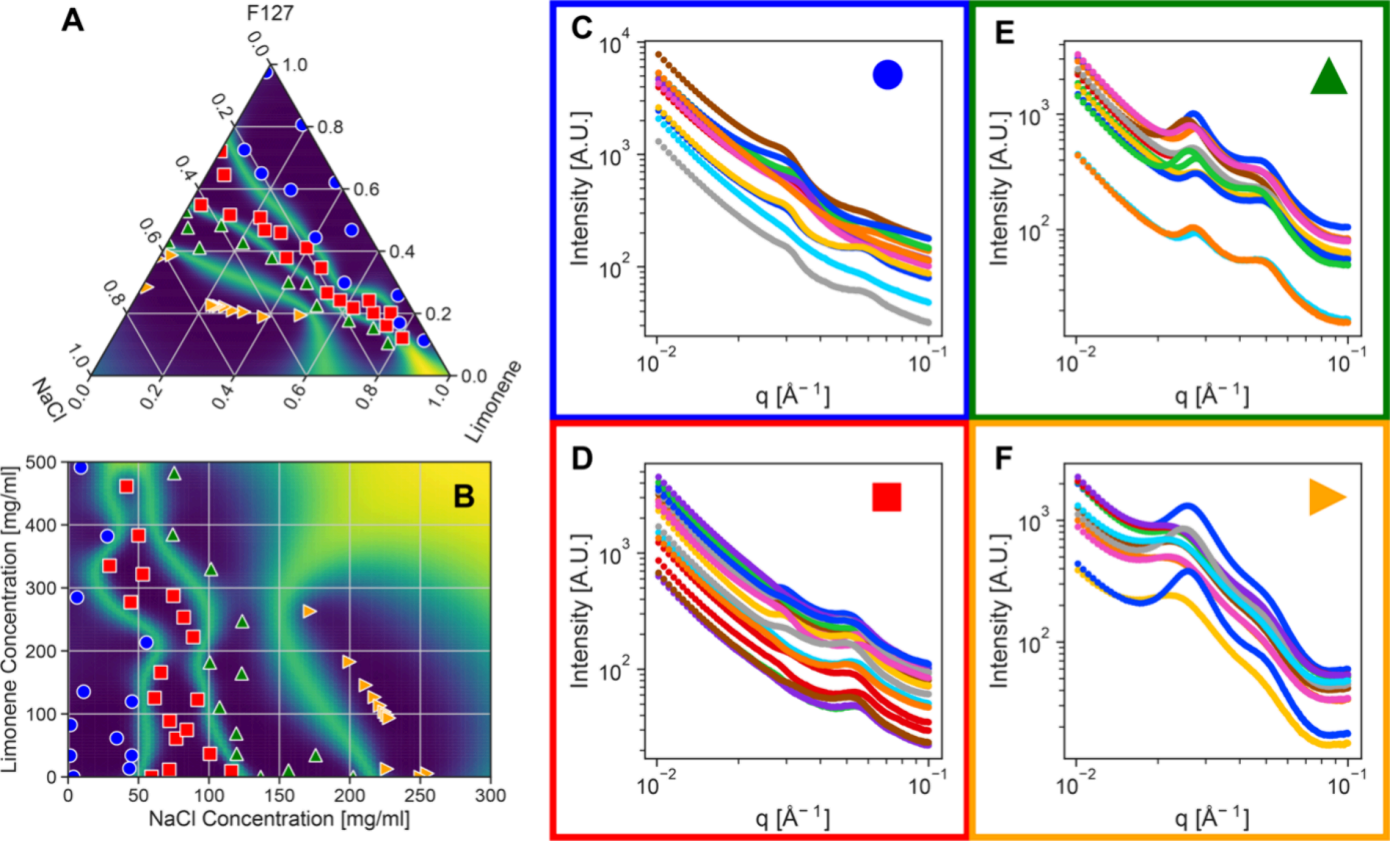
Experimental active learning results on mixtures of Pluronic F127, NaCl, limonene, and water. (A) The final ternary phase diagram measured by the agent and (B) the binary projection of this phase diagram with the symbols denoting the phase identified by the agent and the background colormap, the uncertainty in this phase assignment. Parts C–F show the SAXS data associated with each agent identified phase.

For these solutions, unraveling the phase boundary’s codependence on limonene and salt using conventional scattering experiments with linear grid spacing would be challenging. In the worst-case scenario, we estimate that the grid sampling approach would require as many as 25 x more measurements to achieve the same resolution in boundary location. Furthermore, our *in silico* tests show that the grid approach can miss phases (Figure 3D) and generally poorly identifies the location of boundaries. GP-driven sampling provides a significant improvement over grid sampling as the boundary is only constrained by the Gaussian process kernel and more measurements are conducted near the phase boundaries.

The data sets shown in Figure 4 and Figure 5 exemplify the challenges of studying soft-material phase spaces. While there are similarities to the two systems, the reality of the formulations industry is that precise knowledge of system behavior at specific concentrations, temperatures, and processing conditions is crucial for manufacturability. Manufacturers often want to minimize or maximize certain components to minimize product cost and this can only be achieved with precise knowledge of a phase boundary. Active learning tools, like the one described in this paper, allow industry to rapidly and efficiently map phase spaces.

It is worth pointing out that these first demonstration campaigns used a one-at-a-time sample preparation capability with a typical cycle time of approximately 3 to 5 min depending on sample complexity. This cycle time is well-matched to neutron scattering (with typical measurement times of 15+ min per sample) but not well-matched to pinhole synchrotron SAXS (with typical measurement times below 1 s per sample), with Bonse-Hart USAXS serving as an intermediate case with typically 3 min measurement times. Thus, the direct deployment of one-sample-at-a-time synthesis may not be well matched to pinhole synchrotron SAXS specifically, although there are substantial benefits in reduced material consumption, more reproducible sample preparation, 24/7 operation with lower staffing, remote and multifacility experimentation, reduced downtime to sample changes, etc. Nothing in the agent presented here, however, specifically requires one-sample-at-a-time; indeed, the agent could easily be adapted to either sample from a grid of preprepared samples or dictate the *ex situ* preparation – by robots or humans – of samples for batch measurement using multipoint sampling strategies.

## 5 Conclusion

We have described the design, tuning, and validation of an active learning agent that maps the phase spaces of soft-material formulations using SAS. The agent is designed to be general and does not require *a priori* knowledge of the phase behavior of a system under study. The agent is modular so that, in future studies, it can be optimized for other instruments, to include multiple measurement modalities at once, or to include instrument nonidealities not considered in this work. We tuned the agent through extensive *in silico* experimentation using synthetic data that include real instrument artifacts, including *q*-dependent counting noise, resolution smearing, and stitching artifacts. We showed that this tuning resulted in an agent that is robust to measurement noise and is effective at both small and large numbers of measurements. Finally, we applied the tuned agent to a model formulation problem where we compare the phase maps of two formulations where the difference is whether a petrol- vs naturally derived oil is included in the formulation. The agent primarily identifies a crystallization transition in these formulations, and we show that, through posthoc analysis, the agent could be tuned to identify several unique noncrystalline morphologies if that was the focus of a formulation study.

Active learning agents like the one described here promise to not only revolutionize the way we do measurements but, more broadly, the way we design product formulations. In our current Edisonian world, manufacturers spend significant capital to produce singular, high performance product formulations that minimize production cost, meet regulatory frameworks, and meet the needs of consumers. A highly tuned autonomous platform, such as the AFL, promises to greatly reduce the reformulation time of products leading to a greater number of better, greener choices for consumers. Taking this concept a step further, a fleet autonomous platforms deployed to various local manufacturers could map and optimize hyper-local products that use dynamically or seasonally varying feed streams. While there is significant work to be done toward achieving this goal, our AFL platform advances toward a future with greener, cleaner formulations.

## 6 Experimental Methods

The experimental campaigns in Figures 4 and 5 were conducted on an Autonomous Formulation Laboratory platform, as described in Reference 4, at the Functional Materials Beamline of the Cornell High Energy Synchrotron Source. Pluronic F127, limonene, hexanes, and sodium chloride were purchased from Sigma-Aldrich and used without further purification. For each campaign, six stock solutions were prepared and loaded on the robot deck: F127 (17.1 wt %) in water; NaCl (23.4 wt %) in water; F127 (13.9 wt %) and NaCl (16.8 wt %) in water; F127 (14.6 wt %), NaCl (18.0 wt %), and additive (hexane/limonene) (8.0 wt %) in water; pure water; pure additive (hexanes/limonene). The intermixed stocks (e.g., F127 + additive + salt) were necessary to expand the accessible mixing space and were well handled by the AFL’s mass balance solver.

As described in Reference 4., the samples were prepared via pipetting in an Opentrons OT-2. The preparation sequence and mixing steps (pipet mixing) were fixed for each sample; the use of robotic preparation allows such standardization. While pipetting may not be the final method used to formulate a product, the consistency of the approach enables broadly transferrable results.

The syringe-based AFL sample transfer system was used to load samples into a polyimide flow-through capillary with a diameter of 1.3 mm and rinse the capillary between measurements using water/ethanol mixtures with nitrogen drying. Rinsing of the capillary was varied by measuring the empty capillary in scattering and transmission between each sample.

The scattering measurements were conducted in the beamline’s low divergence mode with a beam size of 250 um x 250 um defined by a series of three slits at a photon energy of 9.8 keV from a single-bounce diamond monochromator. A sample–detector distance of approximately 2 m was used with the precise distance calibrated using silver behenate standards. Data were collected on a Dectris Pilatus 300k pixel-array detector and reduced using the PyFAI integration tool suite before being loaded into the agent. Empty capillary measurements were not subtracted due to large fluctuations in the incident beam intensity and the relatively strong scattering of the samples relative to low instrument background from capillary and windows; we found subtraction with the poor normalization performance introduced more artifacts than it removed.

## Supplementary Material



## Notes

Further discussion and additional figures for labeling pipelines, *in silico* data generation, and description and derivation of the boundary score metric (PDF)
