# Supplementary material for: Autonomous Small-Angle Scattering for Accelerated Soft Material Formulation Optimization
Source: Chem Mater. 2025 Jun 6;37(12):4272–81. doi: 10.1021/acs.chemmater.5c00860 (PMC12199292; doi:10.1021/acs.chemmater.5c00860)
Supplement: Supplementary file 1 [file cm5c00860_si_001.pdf]

Supporting Information for

Autonomous Small-Angle Scattering for Accelerated Soft Material Formulation Optimization

Tyler B. Martin[1,2*], Duncan R. Sutherland[1], Austin McDannald[3], A. Gilad Kusne[3], Peter A. Beaucage[2*]

1. Materials Science & Engineering Division, National Institute of Standards and Technology, Gaithersburg MD, 20899, United States
2. NIST Center for Neutron Research, National Institute of Standards and Technology, Gaithersburg MD, 20899, United States
3. Materials Measurement Science Division, National Institute of Standards and Technology, Gaithersburg MD, 20899, United States

* corresponding authors: tyler.martin@nist.gov and peter.beaucage@nist.gov

Table of Contents

S1 DETAILS OF THE AFL AGENT 3

S1.1 LABELING 4

S1.1.1 DATA PREPROCESSING 4

S1.1.2 SIMILARITY 5

S1.1.3 CLUSTERING 5

S1.2 EXTRAPOLATION 6

S1.3 ACQUISITION 7

S2 DETAILS OF SYNTHETIC DATA GENERATOR 8

S3 *IN SILICO* AGENT TESTING 10

S3.1 TESTING DATASETS FOR LABELING PIPELINE 10

S3.2 LABELING PIPELINE PERFORMANCE WITH SPECTRAL CLUSTERING 11

S3.3 BEST LABELING PIPELINES 12

S3.4 BOUNDARY SCORE DESCRIPTION 13

S3.5 DESCRIPTION OF *IN SILICO ACTIVE LEARNING* 16

S4 EXPERIMENTAL METHODS ..... ERROR! BOOKMARK NOT DEFINED.

S5 REFERENCES 17

S1 Details of the AFL Agent

The processing steps of the AFL agent are schematically shown in Figure S1 and are discussed in detail in the following paragraphs. As our agent relies heavily on open-source implementations of various machine-learning methods, where appropriate, we will broadly describe our usage of a method and provide a reference for the full implementation details of the specific version of the software we used. Our agent codebase `AFL-agent` is open-source and can be downloaded from our usnistgov GitHub repository.[1]

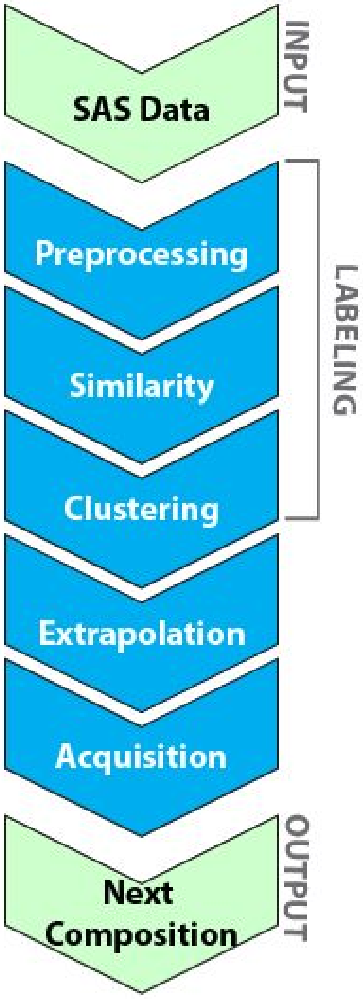

Figure S1: Schematic description of the data processing steps of the AFL agent.

S1.1 Labeling

In this work, the labeling step is composed of a three-step pipeline. The advantage of our labeling approach is that it is very general and can be applied to many material systems and measurements. The downside is that this approach can struggle to distinguish subtle changes in the measurement data or handle continuously changing (2nd order) phase boundaries. Other labeling strategies such as classification provide an attractive alternative but require large amounts of high-quality labeled data. Applying classification approaches to our pipeline is the subject of future work within our program.

S1.1.1 Data Preprocessing

The first step in the agent pipeline is to preprocess, normalize, and correct the provided small-angle scattering (SAS) data. This step is to ensure that the agent can be performant across a variety of materials and instruments. The data is first trimmed to a q-range (domain) that is (a) appropriate for the SAS instrument and (b) contains the feature of interest in the scattering pattern. For the latter, the q-range can be trimmed or segmented to remove features not associated with the physical phenomena of interest in a given study. Next the dataset has all NaN ("not a number") values removed and is back-filled and forward-filled with constant values so that all data is of the same length. This filling of the data is to account for the use of data from different instruments *i.e.* if reference data from one instrument is used to seed a campaign on a different instrument with a slightly different q-range. This is important as some instruments will drop q-values with low signal-to-noise commonly seen at the extrema of a scattering range. Finally, the data is linearly interpolated onto a common, geometrically spaced q-grid. A Savitsky-Golay (SG) filter is applied to lightly smooth each pattern and then calculate the 1st and 2nd derivative.[2] For the SG processing, we typically use a window length of 31 points and a polynomial order (polyorder) of 2.

S1.1.2 Similarity

Next is the similarity calculation. Here, we choose from several similarity metrics and coefficients identified in our *in silico* analysis discussed in Section III of the main text. The most common metrics we use are the Laplacian kernel

$$W\big(I_i(q), I_j(q)\big) = exp\left(-\gamma \|I_i(q) - I_j(q)\|_1\right) \tag{1}$$

and the polynomial kernel:

$$W\big(I_i(q), I_j(q)\big) = \left(\gamma I_i^T(q) I_j(q) + c_0\right)^d \tag{2}$$

Where $I_i(q)$ and $I_j(q)$ are the scattering intensities of measurements i and j as a function of q, γ & c_0 are coefficients used to tune the kernel and d is the power law exponent. We choose a similarity function and apply it to all pairs of data and construct a matrix of similarity values. For all *in silico campaigns* in the main text we used a Laplacian kernel with $\gamma = 0.0025$ applied to both the SG filtered data (0th derivative) and the 1st and 2nd derivatives. We then sum the two similarity matrices and normalize them using the relation:

$$W\big(I_i(q), I_j(q)\big) = \frac{W\big(I_i(q), I_j(q)\big)}{\sqrt{W\big(I_i(q), I_i(q)\big)\, W\big(I_j(q), I_j(q)\big)}} \tag{3}$$

The similarity calculation for the experimental validation was identical except the 2nd derivative was omitted.

S1.1.3 Clustering

With the normalized similarity matrix calculated, we next apply a clustering routine to gather the data into groups. As discussed in Section III of the main text, we have focused on two clustering methods: *spectral clustering* and *Gaussian mixture models*, as implemented in version 1.3.0 of scikit-learn.[3, 4] We use the default parameters with both of these methods except for setting `affinity=”precomputed”` for spectral clustering.

For both clustering methods above, the number of clusters (or phases, N, in our case) must be specified before running the routine. To determine the number of phases, we use a modified silhouette score method as implemented in scikit-learn version 1.3.0.[5] In this method, the Silhouette Coefficient for measurement i is calculated as

$$s_i = \frac{b-a}{\max(a,b)} \tag{4}$$

where a is the average similarity between all of measurements that measurement i belongs to and b is the average similarity between measurement i and the next closest cluster of measurements. To choose the optimal number of clusters, we repeat the clustering and s_i calculations for $N = 2 \ldots 10$ clusters and calculate the mean Silhouette Coefficient, $\bar{S}$, for each. Values of $\bar{S}$ close to 1 indicate a high confidence in the clustering while lower values (with a minimum of 0), indicate lack of confidence in the clustering for that number of clusters. Rather than taking the clustering corresponding to the maximum $\bar{S}_N$, we choose the clustering with the largest *N* that has $\bar{S} > 0.85$. If no clustering satisfies this constraint, the constraint is reduced by 0.05 until a clustering satisfying the constraint is found. If no cluster has $\bar{S} > 0.4$, then we assume that $N = 1$. In our testing, this heuristic approach stabilizes the prediction of the number of phases and produces prediction more consistent with human intuition.

S1.2 Extrapolation

Here, we employ a variational Gaussian process (VGP) classifier as implemented in scikit-learn v1.3.0 and GPFlow version 2.9.0 for the virtual testing and experiments respectively.[6, 7] The VGP allows us to use a non-Gaussian likelihood which is necessary for the implementation of a muti-class classifier.

For the GPFlow implementation of the VGP, we use the Matern32 kernel, RobustMax link function and a MultiClass likelihood.[8-10] The kernel was chosen via an abbreviated version of the *in-silico* tests described in the text, while the link and likelihood functions are recommended choices from the GPFlow documentation. The VGP is fit to the results of the clustering step with the input data being ternary

compositions and the output being the phase label. Once the optimization is complete, the VGP can then predict the mean, $\mu_i(x^*)$, which represents the probability of phase i existing at composition x^* and the posterior uncertainty,$\sigma_i(x^*)$, which is the uncertainty in μ_i at x^*.

For the scikit-learn implementation of the VGP, we used a Matern kernel with `nu=1.5` and an initial length scale of `length_scale=1`. When using this implementation, we use entropy, E, as a stand-in for variance which we calculate from the mean functions for phase i, μ_i, as

$$E = -\sum_i \mu_i \log \mu_i \quad (5)$$

S1.3 Acquisition

Finally, the results of the VGP calculation can be used to choose then next sample to prepare and measure. For the purposes of phase mapping, we use a variance based 'super exploration' acquisition function which modifies the traditional 'pure exploration' approach. The choice of exploration based acquisition works in this case as, due to the construction of the VGP, the uncertainty is guaranteed to be maximized when the probability of multiple phases existing at a composition are equal (*i.e.,* a phase boundary). For pure exploration, we would take the posterior uncertainties calculated from the VGP, sum them to create an overall uncertainty, and then choose the next composition at the point of maximum overall uncertainty. For 'super exploration', we modify the way the composition is chosen from the uncertainty in two ways. First, rather than choosing the maximum uncertainty, we randomly choose a composition from the highest 3-5 % of the uncertainty distribution. Secondly, we introduce a constraint that our selected point cannot be within 1.5 % of an already measured point. These two modifications help alleviate issues with the agent getting 'stuck' and oversampling portions of the phase diagram. Finally, every $n_{density\_sample}$ active learning iterations we switch to an acquisition function that samples based on the point density of measurements rather than the uncertainty. Specifically, we fit a Kernel Density estimation model [11] to the ternary composition values and use this to calculate the log-likelihood of

having measured at given composition. From this, we sample the composition space and randomly choose a position that has a low likelihood, which corresponds to an undersampled portion of the phase diagram. This final modification ensures that we don't place too much trust in the VGP model and that we have measurements that span the available composition range.

S2 Details of Synthetic Data Generator

Our synthetic SAS data generator is built up in two steps. First, a set of compositions and phase labels is gathered from an experiment or by manually tracing the phase boundaries of a figure from the literature. For each unique phase, the alpha shape of the set of points is calculated using version 1.3.1 of the alphashape package.[12] Using this tool, the boundary (*concave hull*) of each phase can be identified for visualization. Most importantly, given an arbitrary point in our composition space, we can iterate over the alpha shapes and find the phase identity of the point.

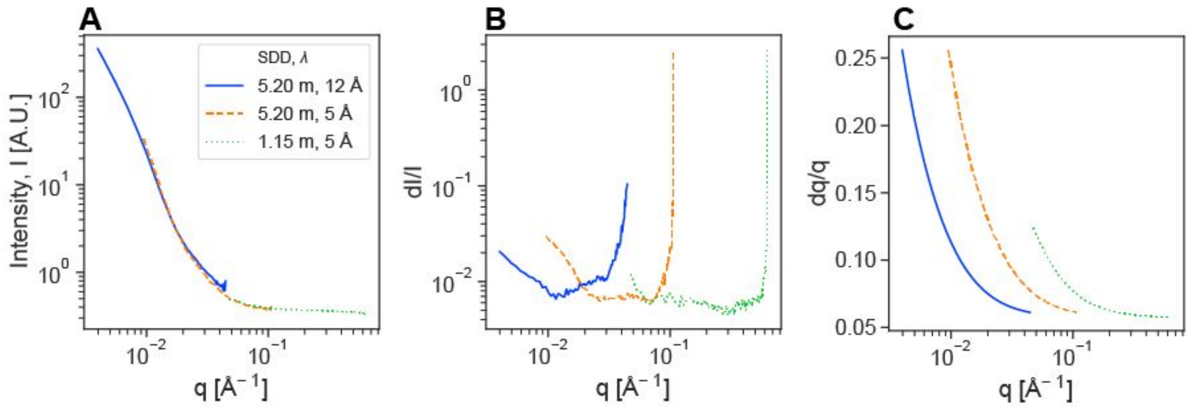

Figure S2: Reference SAS measurements used for introducing resolution, smearing, and counting noise into generated model data. From the measurements, we show the (A) scattering intensity, (B) intensity normalized Poisson noise, $\frac{dI}{I}$, from the 2D area detector propagated through an azimuthal integration, and (C), the q-resolution function normalized by the q-values, $\frac{dq}{q}$. The data were collected on the 10m SANS instrument at the NIST Center for Neutron Research in three configurations corresponding to two sample to detector distances $SDD = 5.2\,m$ and $1.15\,m$ and two wavelengths $\lambda = 12$ Å and 5 Å. The data correspond to a suspension of perfluoronated polymer and carbon nanoparticles. We note that the material identification is done only for completeness as the purpose of these data are just to quantify the instrumentation resolution functions and counting statistics.

The second step is to build the SAS generator for each unique phase. Our approach is to take an analytical model and add in resolution smearing and q-varying counting noise using experimental reference measurements. Our reference data is shown in Figure S2. For the discussion in the main text, our analytical models are chosen from version 1.0.7 of the sasmodels package,[13] although any SAS model generator could be used. The data is resolution smeared by taking the resolution function from reference measurements and convolving it with the model data as described in the SasView documentation.[14] The variable counting uncertainty for each q is calculated by drawing random values from a normal distribution with the mean taken as the measured scattering intensity $I_{expt}(q)$ and the uncertainty $\sigma_{synthetic}$ defined as

$$\sigma_{synthetic}(q) = \sigma_{expt}(q)\left(\frac{\eta}{\left(\frac{1}{N_q}\right)\sum_q\left(\frac{\sigma_{expt}^2\,(q)}{I_{expt}(q)}\right)}\right) \tag{5}$$

In this expression, $\sigma_{expt}(q)$ is the q-dependent noise calculated initially as Poisson noise on the 2D area SAXS or SANS area detector and propagated through the azimuthal integration. The term η is a tuning parameter used to control the level of counting noise in the synthetic data and N_q is the number of q values in the 1-D dataset.

The above approach is an attempt at introducing experimental effects into theoretical models in an efficient, tunable way that avoids expensive Monte Carlo simulations. The inclusion of experimental resolution functions ensures that we don't optimize our agent for unobtainable features of scattering models (e.g., perfect Bessel function fringes). The tunable noise allows us to simulate undercounted measurements which, for the purposes of autonomous learning, might be preferred to maximum the number of compositions that can be sampled. Furthermore, for SAS instruments where data is stitched together from multiple instrument configurations, as is the case with most SANS instruments that are not time-of-flight based, our approach can be iteratively applied. In this mode, the resolution function and counting noise from each configuration is used to generate a synthetic scattering pattern and then these

patterns are stitched together. Importantly, theses stitched curves have the same stitching artifacts that would be present in the real data making our tuning even more relevant to the experimental case.

S3 *in silico* Agent Testing

S3.1 Testing Datasets for Labeling Pipeline

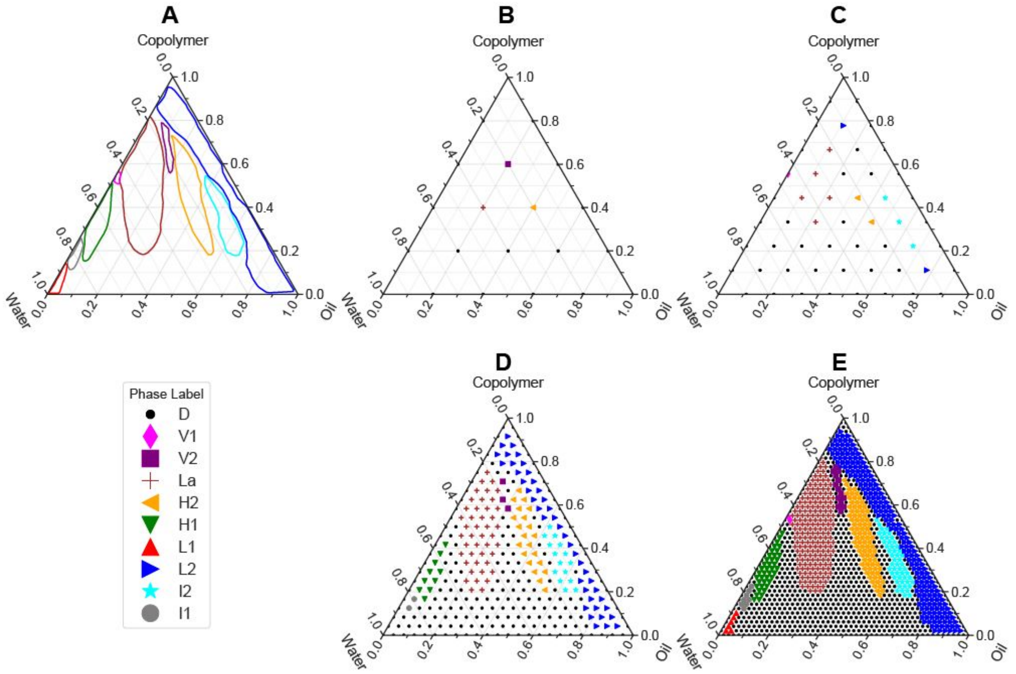

Figure S3: (A) Full phase boundaries and (B-E) discrete datasets for in-silico labeling pipeline testing. Parts (B-E) correspond to $n = 21, 55, 325, and\ 1275$ measurements respectively. The colors and symbols match the legend between Figure 2A and Figure 2B of the main text.

S3.2 Labeling Pipeline Performance with Spectral Clustering

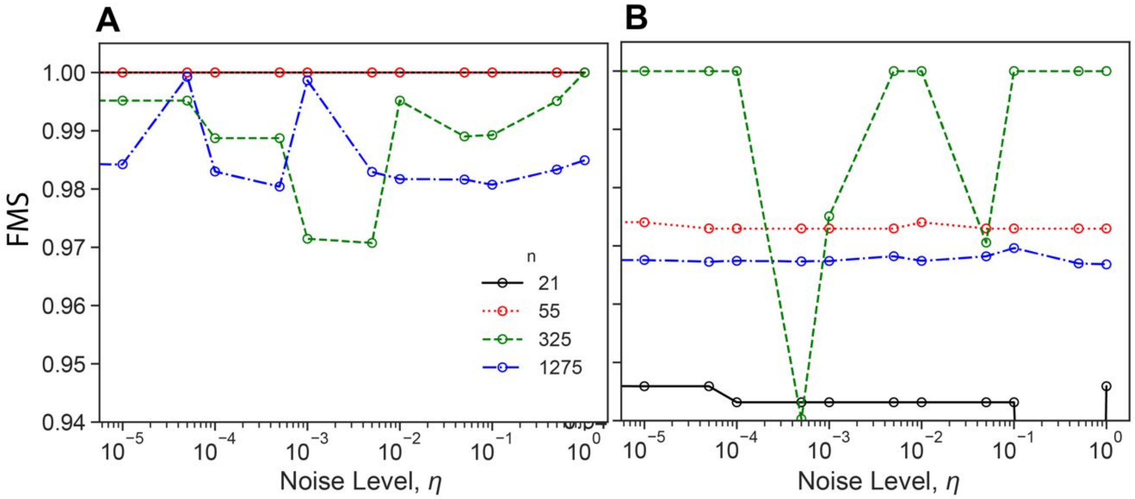

Figure S4: Noise sensitivity of the best labeling pipelines for pipelines using (A) Gaussian mixture models or (B) spectral clustering for the labeling step as a function of the noise level η and number of measurements n.

S3.3 Best Labeling Pipelines

Table S1: Table of the best labelling pipelines identified by in-silico sampling.

n	Clustering Method	Affinity Metric	Distance Matrix?	gamma	degree	c0	co_gamma	FMS
21	gaussian_mixture_model	laplacian	TRUE	0.00001	N/A	N/A	0.0001	1
21	gaussian_mixture_model	laplacian	TRUE	0.0001	N/A	N/A	0.001	1
21	gaussian_mixture_model	laplacian	FALSE	0.001	N/A	N/A	N/A	1
21	spectral_clustering	laplacian	FALSE	0.00001	N/A	N/A	N/A	0.9170
21	spectral_clustering	laplacian	TRUE	0.00001	N/A	N/A	0.00001	0.9219
21	spectral_clustering	rbf	TRUE	0.001	N/A	N/A	0.001	0.9233
55	gaussian_mixture_model	laplacian	FALSE	0.00001	N/A	N/A	N/A	1
55	gaussian_mixture_model	laplacian	FALSE	0.001	N/A	N/A	N/A	1
55	gaussian_mixture_model	laplacian	TRUE	0.001	N/A	N/A	0.00001	1
55	spectral_clustering	poly	FALSE	0.0001	2	100	N/A	0.9732
55	spectral_clustering	poly	TRUE	0.001	2	1000	0.00001	0.9732
55	spectral_clustering	poly	TRUE	0.001	2	1000	0.0001	0.9732
325	gaussian_mixture_model	poly	FALSE	0.00001	1	0	N/A	0.9813
325	gaussian_mixture_model	poly	TRUE	0.00001	1	0	0.00001	0.9815
325	gaussian_mixture_model	poly	FALSE	0.0001	0.5	1000	N/A	0.9899
325	spectral_clustering	laplacian	TRUE	0.05	N/A	N/A	0.05	0.9905
325	spectral_clustering	poly	FALSE	0.00001	2	10	N/A	0.9761
325	spectral_clustering	poly	FALSE	0.00001	4	100	N/A	0.9754
1275	gaussian_mixture_model	poly	FALSE	0.00001	1	1	N/A	0.9869
1275	gaussian_mixture_model	poly	FALSE	0.00001	1	1000	N/A	0.9774
1275	gaussian_mixture_model	poly	TRUE	0.00001	1	0	0.00001	0.9765
1275	spectral_clustering	poly	FALSE	0.00001	3	100	N/A	0.9676
1275	spectral_clustering	poly	FALSE	0.00001	4	100	N/A	0.9677
1275	spectral_clustering	poly	FALSE	0.0001	4	1000	N/A	0.9676

Table S1 shows the results of our brute force sampling of labeling pipelines. From left to right the columns are described as follows. "n" is the number of measurements used in the test and corresponds to Figure S3. "Clustering Method" and "Affinity Metric" are the scikit-learn methods used in the clustering and similarity calculation steps. The "Distance Matrix?" column indicates whether the similarity matrix was multiplied (elementwise) with a Euclidean distance matrix between the compositions of the measurements. The goal was to introduce a locality to the clustering and biases against clusters that span

the composition space. “gamma”, “degree”, & “c0” are the parameters used in the similarity calculation and “co_gamma” is the scaling factor used for the distance matrix (where used). Finally, “FMS” is the Fowlkes-Mallows score as described in the main text.

S3.4 Boundary Score Description

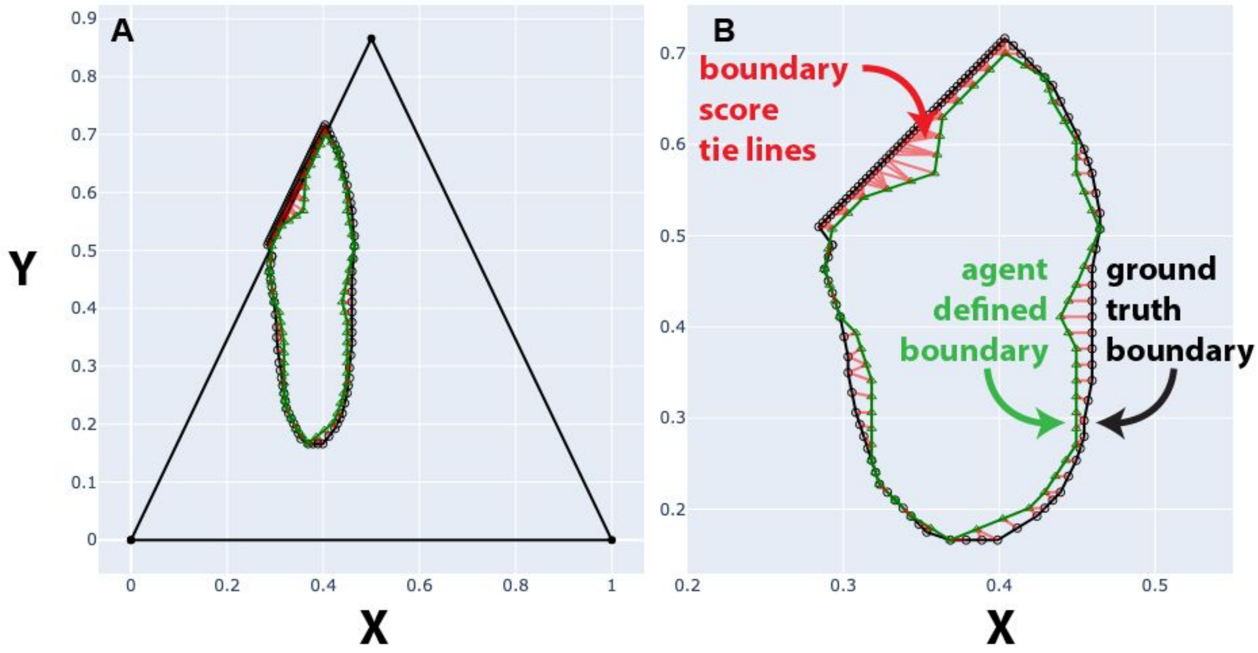

Figure S5: Depiction of the boundary score calculation for the La phase (A) inside the full ternary and (B) with a magnified and labeled view. Both plots are in cartesian coordinates.

A key challenge in benchmarking the performance of the AFL active learning agent is finding an appropriate metric quantify it. Traditional metrics for classification, such as the Fowlkes-Mallows score discussed in the main text, can be applied point by point on a composition grid but this approach is inherently area-biased. This means that the agent can completely misclassify small phases and still produce a high score if the largest phases are mostly correctly identified. Since the primary goal of this agent is to accurately identify the location of phase *boundaries*, rather than identifying phase *regions*, we needed a metric that focused on boundary location rather than area identification.

To address this, we have developed a metric which we call the "boundary score". This metric is calculated as the average distance between the ground truth phase boundary and the agent defined boundary for a given phase in a phase diagram. Briefly, each boundary is found by finding the concave hull for both the ground truth and the agent defined phase regions. Then, for each boundary point in both hulls, the closest boundary point in the opposite hull is found. The average distance between these points of closest approach between the hulls is the boundary score. In Figure S5, the boundary score can be visually understood as the average length of the red tie lines. From this, it should be clear that this metric is explicitly focused on the accuracy of phase boundary placement.

To outline the procedure for calculating the boundary score in more detail: The ground truth boundaries are found by hand-labeling a 5050 point grid of data from the phase diagram found in Reference [15]. Included in these 5050 points is a row of points outside of each side of the ternary boundary. These outside points are forbidden from being selected by the agent during the simulated campaign but are necessary for our "boundaries" to extend to the edges of the ternary during the next step. After the labeled grid is created, we find the boundary definitions using a concave hull calculation as implemented in the `shapely.concave_hull` method with `ratio=0.2`.[16] We then use the `shapely.segmentize` method with `max_segment_length=0.025`.[17] This creates a closed, dense set of points (black circles in Figure S5) which define the phase boundary.

The agent defined phase boundary (green triangles in Figure S5) is calculated similarly. The phase regions are identified by calculating the most likely phase at every composition, x^*, via the VGP derived mean function, $\mu_i(x^*)$ function, described in Section S1. Once the phase regions are identified, we use the same concave hull approach described above to identify the boundary points of each phase.

We now calculate the boundary score between *each pair* of ground truth and agent defined boundaries. This means that *each* agent defined boundary will have M scores corresponding to the M ground truth phases. This is necessary because our clustering approach produces numerical labels that

are unrelated to the physical ground truth labels. Therefore, we must score each agent-defined boundary against all ground-truth boundaries in order to conduct a quantitative matching process. For each boundary node in a given pair of ground truth and agent boundaries, we find the shortest distance to a node in the opposite boundary. After removing any repeated node pairs from the list, the average of these minimum distances is the boundary score for that pair of agent-defined and ground truth boundaries.

Algorithm S1: *Pseudo-code describing how the ground truth label of agent-identified phase boundaries are identified from boundary scores.*

```
n← numerical label of agent identified phases
m← phase label of ground truth phases
B← list of all agent and ground truth boundary label pairs sorted by boundary score (lowest first)
P← empty list to hold agent and ground truth phase boundary pairs
for each pair of boundary labels (m,n) in B do
  if m is not in any pair in P do
    if n is not in any pair in P do
      P← append(m,n)
    end if
  end if
end for
```

At this point, with N agent identified phases and $N \times M$ boundary scores calculated, we identify the “most-likely” ground truth label for each agent boundary using an iterative process described in Algorithm S1. This procedure results in each of the N agent identified phases having a ground truth label and boundary score.

S3.5 Description of *in silico Active Learning*

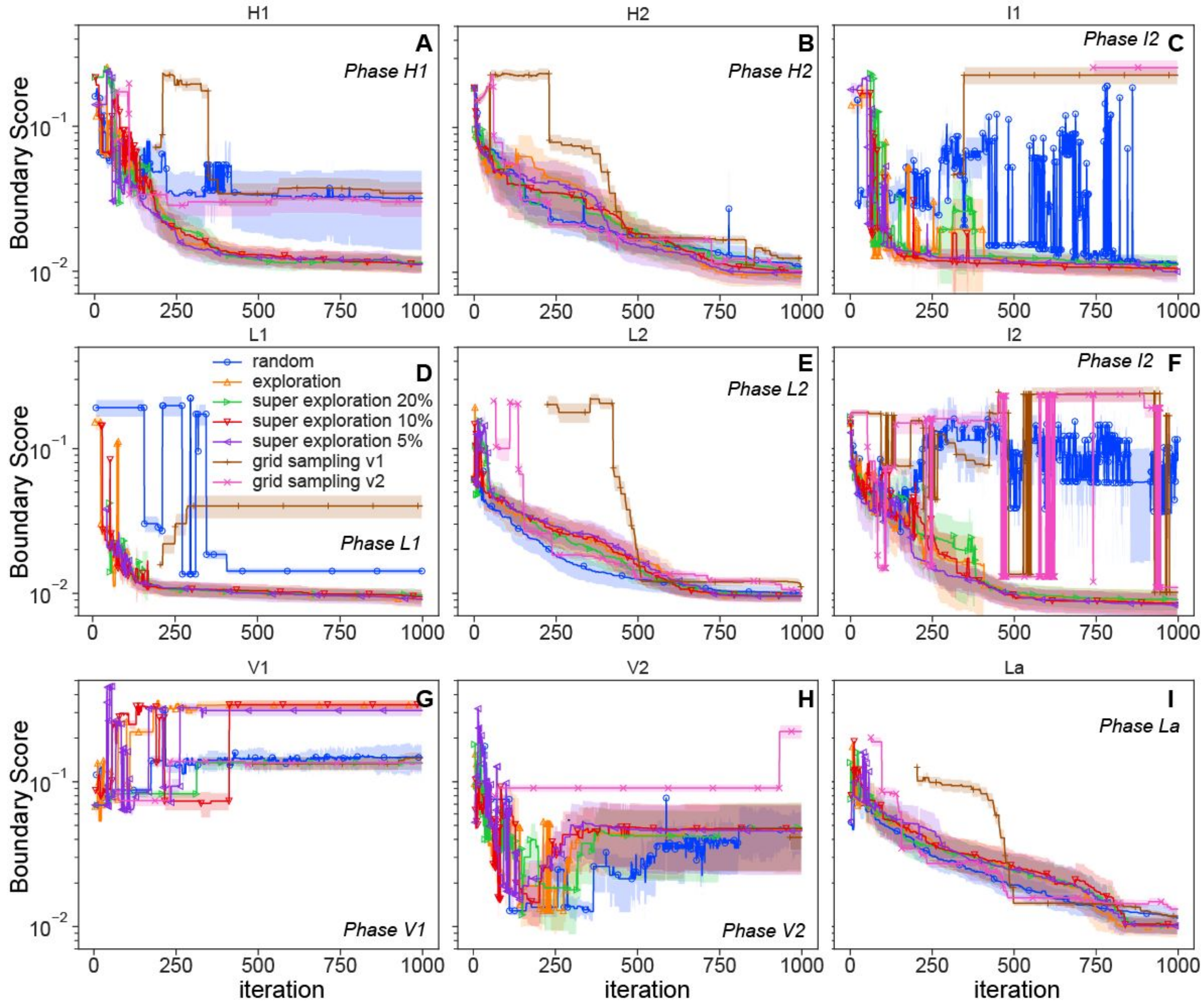

Figure S6: Performance of the AFL agent in full in-silico active learning runs with different acquisition functions (legend in part D) for each phase in Figure 2B. The boundary score is shown as a function of iteration (number of measurements) for each ground truth phase (see subplot titles). The lines and shaded regions represent the mean and standard deviation of seven independent active learning runs from different initial conditions. Where data is not shown for a specific agent and phase, that agent did not identify that phase at that step in any of our virtual campaigns.

Here we briefly describe the acquisition functions (AFs) used in these *in silico* tests:

The “random” AF simply chooses a random point from the selection grid using a uniform distribution, ignoring any input from the labeling or extrapolation step. The “exploration” AF works as described in Section I by randomly selecting a point from the top 5 % of the variance calculated from the VGP. The

“exploration n %” AFs work similarly, except for n % of the steps, the agent randomly chooses a gridpoint without regard to the VGP uncertainty.

For the grid sampling trials, our goal was to mimic how a simple grid scan might be run in an experimental setting. For “grid sampling v1”, first 1000 points are selected from the acquisition function grid. From this grid, the agent sequentially measures points at 0 % copolymer, moving from 100 % to 0 % oil starting. Next, the copolymer content is increased by 5 % and the oil % scan is repeated from 100 % to 5 %. Once 100% copolymer is measured, the agent returns to 2.5 % copolymer, and restarts the oil % scan with steps of 5 % copolymer, skipping over any previously measured rows in copolymer %.

The “grid sampling v2” AF uses similar scans from 100 % to 0 % oil but with varying step sizes in copolymer %. In the first pass, 0 %, 50 %, and 100 % copolymer is measured sequentially with 100 % to 0 % oil scans. Then 20 %, 40 %, 60 %, 80 % copolymer is measured, followed by 10 %, 30 %, 70 %, 90 %. Finally, the stepwise increase in copolymer is done for 5 % steps starting first at 5 % and then 2.5 %, skipping any previously measured rows in copolymer %.

S4 References

1. *AFL-agent repository*. https://github.com/usnistgov/AFL-agent, accessed 2025-04-01.
2. *scipy.signal.savgol_filter*. https://docs.scipy.org/doc/scipy-1.11.2/reference/generated/scipy.signal.savgol_filter.html, accessed 2025-04-01.
3. *sklearn.cluster.SpectralClustering*. https://scikit-learn.org/1.3/modules/generated/sklearn.cluster.SpectralClustering.html, accessed 2025-04-01.
4. *sklearn.mixture.GaussianMixture*. https://scikit-learn.org/1.3/modules/generated/sklearn.mixture.GaussianMixture.html, accessed 2025-04-01.
5. *sklearn.metrics.silhouette_score*. https://scikit-learn.org/1.3/modules/generated/sklearn.metrics.silhouette_score.html, accessed 2025-04-01.
6. *GPFlow*. https://gpflow.github.io/GPflow/2.9.0/index.html, accessed 2025-04-01.
7. *sklearn.gaussian_process.GaussianProcessClassifier. https://scikit-learn.org/stable/modules/generated/sklearn.gaussian_process.GaussianProcessClassifier.html, accessed 2025-04-01.*
8. *gpflow.kernels.Matern32*. https://gpflow.github.io/GPflow/2.9.0/api/gpflow/kernels/index.html#gpflow-kernels-matern32, accessed 2025-04-01.

9. *gpflow.likelihoods.RobustMax*. https://gpflow.github.io/GPflow/2.9.0/api/gpflow/likelihoods/index.html#gpflow.likelihoods.RobustMax, accessed 2025-04-01.
10. *gpflow.likelihoods.MultiClass*. https://gpflow.github.io/GPflow/2.9.0/api/gpflow/likelihoods/index.html#gpflow.likelihoods.MultiClass, accessed 2025-04-01.
11. *sklearn.neighbors.KernelDensity*. https://scikit-learn.org/1.3/modules/generated/sklearn.neighbors.KernelDensity.html, accessed 2025-04-01.
12. *Alphashape Toolbox*. https://github.com/bellockk/alphashape/releases/tag/v1.3.1, accessed 2025-04-01.
13. *Sasmodels 1.0.7*. https://github.com/SasView/sasmodels/releases/tag/v1.0.7, accessed 2025-04-01.
14. *SasView Resolution (Smearing) Functions*. https://www.sasview.org/docs/user/qtgui/Perspectives/Fitting/resolution.html, accessed 2025-04-01.
15. Alexandridis, P., U. Olsson, and B. Lindman, *A Record Nine Different Phases (Four Cubic, Two Hexagonal, and One Lamellar Lyotropic Liquid Crystalline and Two Micellar Solutions) in a Ternary Isothermal System of an Amphiphilic Block Copolymer and Selective Solvents (Water and Oil).* Langmuir, 1998. **14**(10): p. 2627-2638.
16. *shapely.concave_hull*. https://shapely.readthedocs.io/en/2.0.2/reference/shapely.concave_hull.html, accessed 2025-04-01.
17. *shapely.segmentize*. https://shapely.readthedocs.io/en/2.0.2/reference/shapely.segmentize.html, accessed 2025-04-01.
